# Cuproptosis-related lncRNAs and genes: Potential markers for glioblastoma prognosis and treatment

**DOI:** 10.1371/journal.pone.0315927

**Published:** 2025-02-06

**Authors:** Yajia Chen, Jingxian Zhang, Weiqian Zheng, Hongwu Xu

**Affiliations:** 1 The Tenth Affiliated Hospital, Southern Medical University (Dongguan People’s Hospital), Dongguan, Guangdong Province, China; 2 Shantou University Medical College, Shantou, Guangdong, China; Alexandria University, EGYPT

## Abstract

Despite the availability of various treatment options, glioblastoma (GBM) remains an extremely aggressive form of glioma with a poor prognosis. In recent studies, regulatory cell death (RCD) has been identified as an effective mechanism to suppress glioma. Cuproptosis, caused by intracellular copper, is a novel RCD process that affects chemotherapy efficacy and glioma progression; however, the precise function of cuproptosis-related lncRNAs (CRLs) and cuproptosis-related genes (CRGs) in GBM remains uncertain. To determine whether CRLs and CRGs have prognostic significance, a GBM cohort in TCGA to build a novel cuproptosis-related risk model. Two high-risk CRLs (AC091182.2, AC005229.4) and their co-expression CRGs (*LIPT2*, *GLS*) were identified and verified to constitute an independent prognostic indicator of GBM. RT-qPCR analysis confirmed that the high-risk CRLs and CRGs were highly expressed in GBM cells compared to normal astrocytes. By constructing a mouse GBM model, high-risk CRLs and CRGs were found to be expressed at higher levels in tumor tissues. Furthermore, to verify whether these CRLs and CRGs are associated with GBM cuproptosis, cuproptosis cell models were constucted in GBM cell lines and astrocyte by using Elesclomol and CuCl_2_. It was found that the expression of high-risk CRLs and CRGs was decreased upon cuproptosis-induced in GBM cells. Interestingly, normal astrocytes were less sensitive than GBM cells to cuproptosis-inducing drugs, and the effects of the drugs on the expression of the CRLs and CRGs in normal astrocytes were opposite to that of in GBM cells. In conclusion, by constructing a novel cuproptosis-related risk model, two high-risk CRLs and CRGs were identified. Their specific pointing to GBM has been demonstrated through a variety of experiments. These CRLs and CRGs might serve as prognostic markers and indicators for GBM and provide theoretical support for future GBM treatment.

## Introduction

Gliomas are the most prevalent and highly malignant form of primary brain tumors [1]. Among the four categories of glioma, glioblastoma multiforme (GBM, WHO stage IV) is the most fiercely aggressive molecular subtype [2]. Despite the availability of various treatment options, including surgery and chemoradiotherapy, the prognosis of patients with GBM remains poor [3–5]. Furthermore, in molecular therapy, many agents that target classical signaling pathways related to cancer have been identified, but the therapeutic effects on GBM have been modest, and clinical trials are still in progress [6]. Therefore, it is urgent to find more effective targeted molecular strategies for the treatment of GBM.

In humans, the brain is the principal site of copper accumulation subsequent to the liver [7–9]. In adults, about 95% of copper binds to the brain choroid plexus and other major copper-binding proteins [10]. The concentration of copper in cerebrospinal fluid (approximately 70-80 μM) is higher than in other regions of the human body, which enhances the likelihood of specific copper signaling in the brain [11,12]. Copper ions at physiological concentrations exert a neuroprotective role and are essential for the maintenance of normal physiological functions in the nervous system [13]. However, disruption of intracellular copper ion homeostasis is directly associated with the progression of various neurological disorders. Copper deficiency can lead to cerebral atrophy, degeneration of gray and white matter, reduction in neuronal count, and stagnation of mental development, among other pathologies [14]. Also, excessive intracellular accumulation of copper can induce oxidative stress and perturb cellular functions, subsequently leading to pathologies, such as cancer [15,16]. Furthermore, Tsvetkov et al. have identified that excessive copper ions trigger regulated cell death (RCD), and have termed this form of RCD as “cuproptosis” [17]. In summary, copper homeostasis plays a crucial role in maintaining physiological activities of cells, including tumor cells, and is subject to stringent regulation. Therefore, disrupting copper homeostasis in GBM can induce cuproptosis, thereby limiting its progression and development.

Cuproptosis, first described by Tsvetkov et al., is a unique form of RCD characterized by Cu-dependent cytotoxicity [17,18]. It is a controlled mode of cell death that is distinct from other type of RCD and is mediated by an ancient mechanism: protein acylation [17,18]. Cuproptosis can be inhibited by copper ion carriers and copper chelators, but not by other cell death inhibitors [17,18]. Previous research has reported that progression of gliomas is associated with copper-dependent death [19]. Furthermore, cuproptosis is modulated by specific signaling factors and pathways that could potentially serve as targets for GBM treatment [20,21]. It has been reported that the cuproptosis gene FDX1 is a prognostic biomarker that is associated with immune infiltration in glioma [22]. However, it remains unknown which cuproptosis-related factors play a dominant role in the development of GBM. In recent studies, RCD is regulated by specific lncRNAs and genes, which indicates potential for targeting as a specific approach to cancer therapy. For example, CST1 has been demonstrated to regulate GPX4 protein stability via OTUB1 to inhibit ferroptosis and promote gastric cancer metastasis [23]. Furthermore, lncRNA MT1DP loaded by folate-modified liposomes sensitizes erastin-induced ferroptosis by regulating the miR-365a-3p/NRF2 axis in non-small cell lung cancer [24]. This indicates that targeting RCD-specific lncRNAs and co-expressed genes may serve as a specific therapeutic approach for cancer treatment. Thus, targeting key cuproptosis-associated genes and their co-expressed lncRNAs may provide a specific therapeutic approach for inducing cuproptosis in GBM cells, thereby limiting tumorigenesis and progression, and enhancing therapeutic sensitivity, offering a novel avenue for GBM treatment.

## Materials and methods

### Data acquisition

From the TCGA GBM dataset (https://cancergenome.nih.gov/), GBM transcript data, clinical information, and mutation data were obtained for 19,938 mRNA samples, 16,876 lncRNA samples and 599 clinical samples (230 female and 390 male) by Perl scripts (Strawberry Perl (64-bit) 5.30.0.1-64bit). The dataset provided details such as age, gender, survival status, and survival time, but not the grade, which was unspecified. Initially, we obtained mRNA expression data of 19 cuproptosis-related genes from 19,938 mRNA samples. The 16,876 lncRNA samples and CRGs’ mRNA samples were preprocessed. The lncRNA samples with expression values less than 0.1 were removed, and normal samples were deleted. The CRGs’ mRNA samples were subjected to the same treatment. In order to obtain cuproptosis-related lncRNAs, a Pearson correlation analysis employed and visualized between 16,877 lncRNA samples and 19 CRLs mRNA expression data by using “limma”package (v.3.54.2), tidyverse” (v.2.0.0, “ggplot2” (v.3.4.2) and “ggExtra” (v.0.10.0) with a threshold of | Pearson | >  0.5 and p <  0.05. Finally, 853 cuproptosis-related lncRNAs from 16,877 lncRNA samples were identified. To identify clinical samples associated with cuproptosis-related lncRNAs, 599 clinical samples and 853 cuproptosis-related lncRNAs were merged using Perl scripts and the R “caret” package (v.6.0-94). Then, through “glmnet” (v.4.1-7), “survminer” (v.0.4.9) and “caret” (v.6.0-94) R packages, univariate Cox regression analysis was performed to identify clinical samples with risk significance. P-value < 0.05 was used as a threshold for filtering. Finally, 159 clinical samples with risk significance and associated with cuproptosis were identified. And 159 clinical samples were included and divided randomly into a training set (n = 80) and test set (n = 79).

### Identification of CRLs and construction of a risk score model

By univariate Cox analysis, we identified 19 CRLs from 159 clinical samples in the training set with p-value < 0.05. Using the “glmnet” (v.4.1-7), “survminer” (v.0.4.9), “survival” (v.3.5-5) and “caret” (v.6.0-94) R packages, we employed Lasso-Cox regression with p-value < 0.05 to eliminate non-informative lncRNAs, ultimately incorporating 7 CRLs. Next, we utilized multivariate Cox proportional hazards regression with p-value < 0.05 to identify 3 CRLs and their coef value. We calculated the risk score for each patient based on these 3 CRLs’ coef value and constructed a prognostic risk model.

The training set and testing set were divided into high-risk (risk value > median) and low-risk (risk value <median) groups according to the CRLs median risk score (Median all clinical samples = 1.005904127, Median testing set =  1.024668706, Median training set =  1.00561753). The risk score was calculated as follows:

Risk score =  (0.382424336868827 × AC091182.2 expression level)  +  (0.893936167468944 × AC005229.4 expression level) +  (  − 0.658336798818582 × ZNF22-AS1 expression level).

### Evaluation of independent prognostic factors and development of the nomogram

Receiver operating characteristic (ROC) analysis was utilized to evaluate the accuracy and sensitivity of our model, which was based on the predictive significance of CRLs. The “survival” (v.3.5-5), “survminer” (v.0.4.9) and “timeROC” (v.0.4) R packages were used to draw ROC curves. To assess model feasibility, we performed Kaplan-Meier analysis on the high- and low-risk groups across all data sets using the “survival” (v.3.5-5) and “survminer” (v.0.4.9) R package [18]. A variable was deemed an independent prognostic factor only if it demonstrated a p-value < 0.05 in both univariate and multivariate Cox regression analyses. Furthermore, Kaplan-Meier analysis was employed to reveal disparities in progression-free survival (PFS) between the high- and low-risk groups within the training set. The “survival” (v.3.5-5) and “RMS” (6.6-0 version) software packages were used to develop a nomogram that integrates clinical and pathological factors with the risk score according to the 1, 3- and 5- year survival rates of GBM patients. Then, ROC analysis was performed to evaluate the accuracy and sensitivity of nomogram.

### Cell culture

The human GBM cell lines U87 (Hunan Fenghui Biotechnology Co., Ltd), T98G (Fuheng, CO., Ltd), LN229 (Se Ou Biology CO., Ltd), and U343 (MeilunBio, CO., Ltd); the human normal astrocyte cell line SVGP12 (Hunan Fenghui Biotechnology Co., Ltd); and the mouse GBM cell line G422-GFP-LUC (Hunan Fenghui Biotechnology Co., Ltd) were cultured in Dulbecco’s modified Eagle’s medium supplemented with 100 u/mL penicillin and streptomycin and 10% fetal bovine serum. The cells were placed in a 37°C environment with humidified atmosphere of 5% CO_2_.

### Establishment of a GBM xenograft mouse model

This study was carried out in strict accordance with the recommendations in the Guide for the Care and Use of Laboratory Animals of the National Institutes of Health. The protocol was approved by the Laboratory Animal Ethics Committee of Shantou University Medical College (Protocol Number: SUMCSY2024-001). All surgery was performed under Tribromoethanol anesthesia and Meloxicamum, and all efforts were made to minimize suffering. The mice were group-housed in the animal center facility of Shantou University Medical College. All personnel performing animal experiments have completed training in laboratory zoology, including animal care or handling.

Twenty adult female BALB/c mice (18–22 g) were purchased from Guangdong Sijia Jinda Biotechnology Co. Ten of them were pre-experimental. Briefly, adult mice were anaesthetised with Tribromoethanol and removed head hair, and fixed on a brain stereotaxic apparatus. The scalp was cut along the middle suture and a burr hole was drilled in the skull. The G422-GFP-LUC GBM cell line (1 x 10^6^) suspended in Dulbecco’s modified Eagle’s medium was injected stereotactically through a 10 μl Hamilton syringe (Hamilton, MA, USA) to a depth of 2 mm. The cell suspension was delivered at a rate of 0.5 μl/min. After implantation of 3 μl of cells, the needle was left in place for 10 minutes before withdrawal. Their biological behaviors were examined every two days after the operation, including body weight changes, abnormal behaviors (e.g., uni-directional circling, deliberate scratching of the face, and aggravation of the stress response), fur ruffles, and reduced mobility. Tumor growth was observed using a Small Animal In Vivo Imaging system, IVIS (IVIS Kinetic, USA) after a single intraperitoneal injection of 0.2 ml/g of fluorescein potassium salt (15 mg/ ml in PBS, ST196, Beyotime, Shanghai, China) for 10 min on pre-injection and postoperative days 7 and 14. When the tumor luminescence intensity was in the range of 2 ~  4e + 05, the model mice were dissected. It was found that the tumors generally grew up to the boundaries of the range of tumor volumes specified in the animal ethic (tumor diameters not exceeding 1 cm). In subsequent feeding, we found that mice died around 20 days after injection, and the mean death time for all mice was 25 days. Therefore, the remaining 10 mice were cared for and observed after brain stereotaxic injection of GBM-G422-GFP-LUC cells. After the 14th day of injection, the model mice were euthanized with Tribromoethanol anesthesia and Meloxicamum, and were perfused with saline solution through the left cardiac ventricle and sacrificed. Then, the location of GBM in brain tissue was determined by HE-staining and pathological imaging. The tumor tissues in the surgical group and the normal brain tissues in the control group were evaluated.

### The correlation between the risk score and immune status

Single-sample gene set enrichment analysis (ssGSEA) with the Gene Set Variation Analysis (GSVA) R package (V.1.40.1) was used to evaluate the immune cell infiltration in a publicly available GBM dataset [25]. Relative expression values were displaying by heat mapping. CIBERSORT, a method based on linear support vector regression, was developed to evaluate the proportions of 22 immune cell types represented by bulk tumor sample expression data [26]. CIBERSORT by “e1071” (v.1.7-13), “limma” (v.3.54.2) and “preprocessCore” (v.1.60.2) were applied to TCGA-GBM RNA-Seq data to determine the immune cell abundance, and CRLs in immune cells were analyzed by Spearman correlation. Using the ESTIMATE algorithm (“estimate” (v.1.0.13) R package), we evaluated the immune, stromal, and ESTIMATE scores across all GBM samples and conducted differential analyses between high- and low-risk groups. The results were visualized using “reshape2” (v.1.4.4) and “ggpubr” (v.0.6.0) R packages.

### Establishment of the GBM cuproptosis cell model

According to the research on cuproptosis-inducing drug (Elesclomol and CuCl_2_) [17–19,27], concentration gradients of Elesclomol (10 nM, 20 nM, 40 nM) (GC13885, GLPbio, Shanghai, China) and CuCl_2_ (1 μM, 5 μM, 10 μM) were administered to GBM cell lines (AAPR732-100, Pythonbio, Guangdong, China). The cell density and state were observed at 0 h, 1 h, 2 h and 3 h after drug administration. The optimal concentrations that made the cells round, crumple, and rupture 2 h after administration (10 μM CuCl_2_ and 40 nM Elesclomol) were taken as the optimal concentration (S1 Fig).

### Cell viability assay

The Cell counting kit-8 (BMU10-CN, Abbkine, Wuhan, China) was used to assess cell viability. Cells (1 ×  10^4^ per well) were inoculated into 96-well plates. After treatment with 10 μM CuCl_2_ and 40 nM Elesclomol for 2 hours, the indicated reagents were added. A 10 μL CCK-8 solution was added to each well, and the cultures were incubated at 37°C for 2 hours. Absorbance at 450 nm was measured and quantified by an enzyme marker (Biotek Elx800, USA). Three replicate wells were tested for each group in each experiment, and at least three independent experiments were performed. The absorbance of each group was normalized by the blank group (without any treatment of the indicated cells).

### Western blot analysis

Cuproptosis-treated cells were collected and lysed on ice in RIPA buffer containing 1 mM protease inhibitor and phosphatase inhibitor for 40 min, followed by centrifugation at 12,000 rpm for 30 min at 4°C. The supernatants were collected and quantified by BCA protein assay (P0009, Beyotime, Shanghai, China). Protein (15–30 μg) was loaded onto SDS-PAGE gels and electrophoresed at 80 V for 15 min, followed by electrophoresis at 120 V for 40 min. The proteins were then transferred at 270 mA to a 0.22 μm PVDF membrane (Millipore, Tullagreen, Carrigtwohill, Ireland) for 72 min. The membranes were incubated with primary antibodies (FDX1, Abmart, T510671S, 1:1000; β-Tubulin, Abclonal, AC008, 1:1000) overnight in TBST containing 5% milk powder. Immunoblot imaging was performed using HRP-coupled secondary antibodies (HRP-labeled Goat Anti-Rabbit IgG (H + L), Beyotime, A0208, 1:1000) and ultra-high sensitivity ECL (BL520B, Biosharp, Anhui, China) with the MIni Chemi610 imager (SINSAGE, Beijing, China). The membranes were clipped to the appropriate target protein range prior to antibody incubation, and the strips were spliced back to the intact membrane state for exposure. Images of the pre-cut membranes and whole exposure membranes are shown in S1 Raw Images.

### Quantitative real-time PCR analysis of high-risk CRLs and co-expressed CRGs

RNA from human GBM cell lines, the GBM cuproptosis cell model and the GBM mouse model was extracted with Trizol (Invitrogen, USA) according to the manufacturer’s protocol. Reverse transcription was conducted using a HiScript II 1st Strand cDNA Synthesis Kit (+gDNA wiper) (Vazyme) containing random and oligo dT primers to obtain cDNA. RT-qPCR was performed on an ABI 7500 Real TimePCR instrument with ChamQ Universal SYBR qPCR Master Mix (Vazyme). A three-way replication experiment was performed in triplicate in three independent experiments, and ACTB was used as the internal control. S2 Table lists the sequences of all primers used.

### Data analysis

For analysis, R statistical programming language (R v.4.2.3) and Perl (Strawberry (64-bit) 5.30.0.1-64bit) were used. The log-rank test was utilized to generate Kaplan-Meier curves. The expression levels of CRLs and CRGs were evaluated in high-and low-groups by using the T-test. Pearson correlation analysis with a threshold of | Pearson | >  0.5 and p <  0.05. Additionally, univariate and multivariate Cox regression analysis with a threshold of P-value < 0.05 were used to investigate independent prognostic indicators for OS based on CRLs. Lasso-Cox regression with a threshold of P-value < 0.05; multivariate Cox regression analysis with a threshold of P-value < 0.05. The correlation matrix was prepared according to the Spearman test. P-values were two-sided and < 0.05 was considered significant. In the experimental section, all experimental data were normalized and comparisons between the two groups were conducted using the Student’s t-test. P-values were one-sided and < 0.05 was considered significant. The session information of R is shown in S2 Fig.

## Results

### Identification of CRLs and co-expressed CRGs with prognostic significance

**Fig 1:**
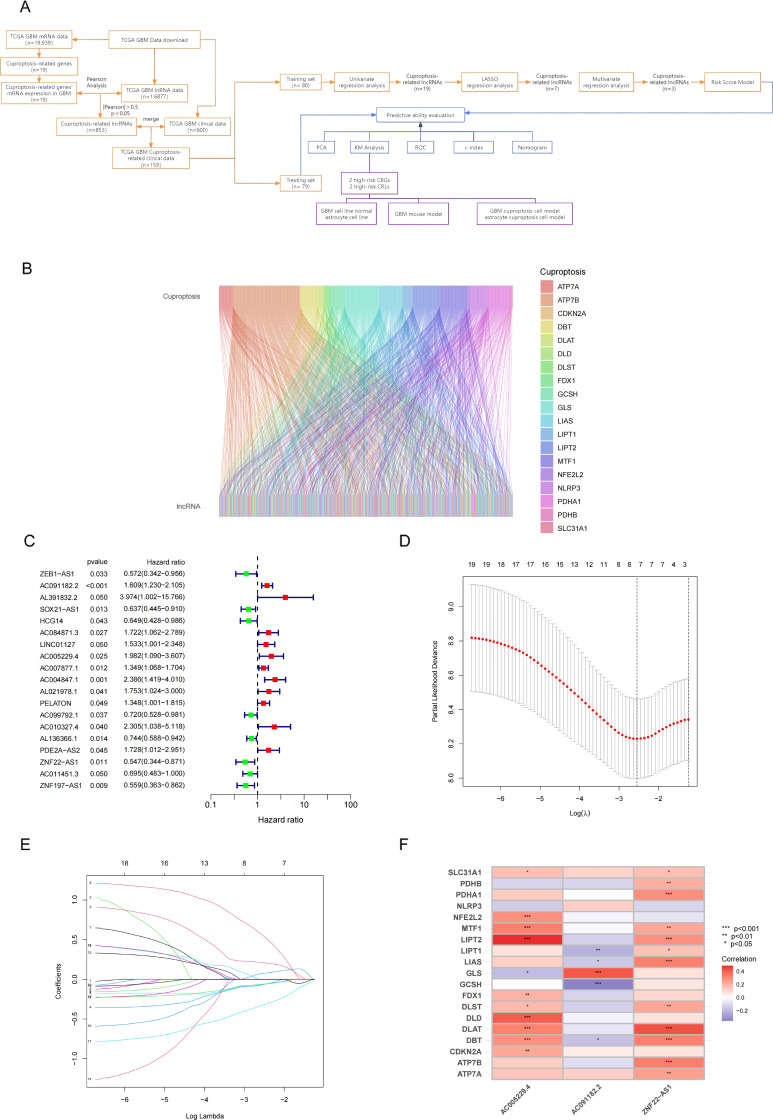
Data processing and predictive model construction. (A) The workflow of this study. (B) The Sankey diagram shows the connection degree between the 19 cuproptosis-related mRNAs and the 853 lncRNAs. (C) Univariate Cox regression analysis. Green indicates low risk and red indicates high risk lncRNAs. (D, E) Lasso Cox regression analysis (lasso Lambda and lasso Cvfit). (F) Correlation between lncRNAs involved in model construction and cuproptosis-related genes.

Fig 1A provides an overview of our study design. Based on a study by Tsvetkov et al. [17,18], 853 CRLs were identified by Pearson’s correlation analysis between 19 CRGs’ mRNA samples and 1,6877 lncRNA samples from TCGA GBM dataset (Fig 1B). We simultaneously downloaded 599 GBM clinical samples (230 female and 390 male) in TCGA (S1 Table), and then surveyed the 599 clinical samples to access the expression of the 853 CRLs in clinical samples with univariate Cox regression analysis. Finally, 159 clinical sample (training set, n = 80; testing set, n = 79) with risk significance were identified. A predictive model was created through the training set, and the testing set was used to validate the accuracy of the model.

To identify CRLs associated with prognosis, we performed univariate Cox hazard, LASSO regression and multivariate Cox analyses (Fig 1C–E). Finally, 3 CRLs with prognostic significance (AC005229.4, AC091182.2, ZNF22-AS1) were identified. Their coef value used to calculated risk score and build the prediction model (S3 Table).

The co-expression of corresponding CRG is shown in the heatmap (Fig 1F). According to Fig 1D, *LIPT2*, *GLS* and *DLAT* were identified as the CRGs that were most highly co-expressed with AC005229.4, AC091182.2 and ZNF22-AS1, respectively.

### Assessment of the prognostic potential of the new predictive model

**Fig 2:**
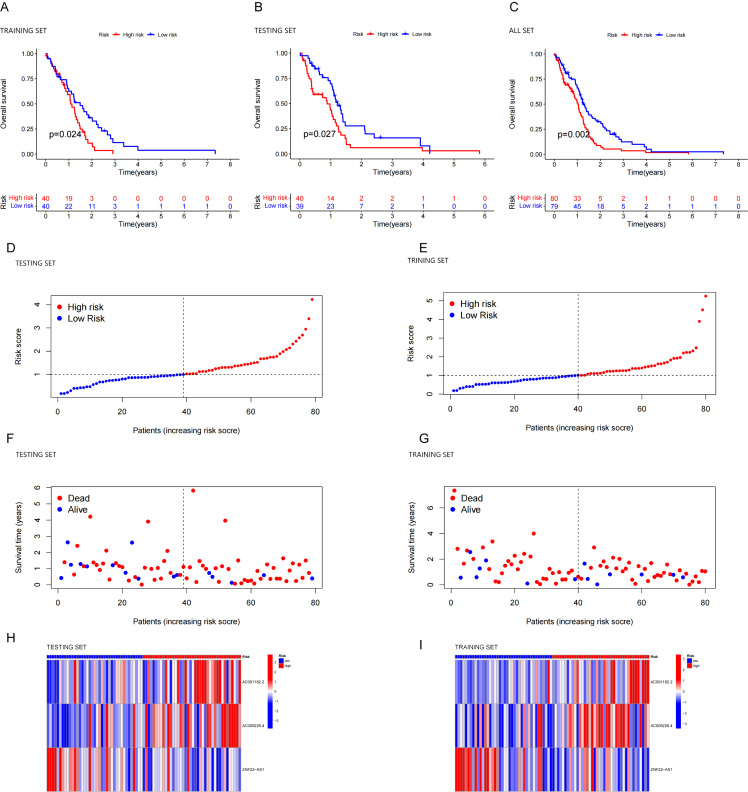
Evaluation of risk model. (A–C) Kaplan–Meier curves for survival analysis in the high- and low-risk groups. The Kaplan-Meier survival curves of the high and low risk groups in the training set (A, p = 0.024 < 0.05), the testing set (B, p = 0.027 < 0.05) and the combined set (C, p = 0.002 < 0.01). (D, E) Risk score distribution in patients with GBM. (F, G) Survival status in patients with GBM. (H, I) Heatmap of prognostic markers and overall survival.

According to the median risk score, we divided the patients into high- and low-risk groups Kaplan-Meier analysis was performed to compare the overall survival between high-risk and low-risk group. The results showed that the high-risk group had noticeably inferior OS compared to the low-risk group across the training set (Fig 2A, p = 0.024 < 0.05), testing set (Fig 2B, p = 0.027 < 0.05), and combined set (all the clinical samples) (Fig 2C, p = 0.002 < 0.05). The risk score and survival status of the training and testing group each verified that the mortality rate increased with higher scores (Fig 2D–G). Furthermore, for the training and testing set, the expression patterns of the CRLs were similar in the low-and high-risk groups (Fig 2H, I). The above results show that the predictive performance of the model is good. According to our predictions, higher expression of AC091182.2 and AC005229.4 were linked to shorter survival times, increased mortality and higher risk scores, while ZNF22-AS1 exhibited the opposite correlation. These results confirm the predictive performance of the model and indicate that AC091182.2 and AC005229.4 are high-risk CRLs, while ZNF22-AS1 is a low-risk CRL.

### The CRL‐based model is an independent prognostic factor for GBM patients

**Fig 3:**
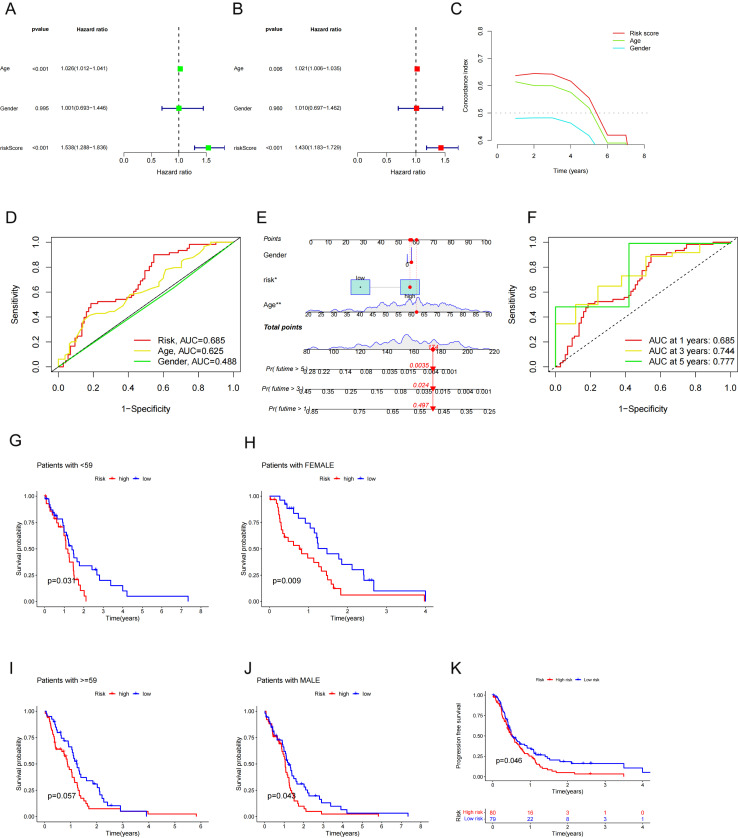
Analysis of independent prognostic factors. (A) Forest plots showing univariate Cox regression analyses of the combined set. (B) Forest plots showing multivariate Cox regression analyses of all samples. (C) C-index demonstrating that the predictive accuracy of the risk model is superior to that of other clinical parameters. (D) ROC analysis demonstrating that the predictive accuracy of the risk model is superior to that of other clinical parameters. (E) Nomogram based on risk and clinical factors to predict the 1-, 3- and 5-year survival of GBM patients. (F) TimeROC curve prediction of the 1-, 3-, and 5- year OS of GBM patients. (G-J) Stratification analysis of the risk score in GBM: (G, I) Age (<59 and>  = 59 years); (H, J) Gender (female and male). (K) Kaplan–Meier curves of progression-free survival (PFS).

To further evaluate the reliability of the CRL-based model as a predictive marker for GBM, we conducted Cox regression analysis of all clinical samples (n = 159). Significant variations were found in the risk score between different age groups according to univariate Cox analysis (Fig 3A). Multivariate Cox analysis showed that both the age (HR =  1.430, 1.183–1.729, p <  0.001) and risk score (HR =  1.430, 1.183–1.729, p <  0.001) estimated by the CRL‐based model were independent predictive variables (Fig 3B). Both the C-index and the ROC curve suggested that the prediction accuracy of the prognostic model was much higher than that of other clinical factors, including age and gender (Fig 3C, D). To predict the 1-, 3- and 5-year survival rates of GBM patients, we created a nomogram that incorporates gender, age and risk scores by integrating clinical and pathological factors with the risk score (Fig 3E). The CRL-based model showed good accuracy and diagnostic value for OS, with ROC curves yielding AUCs of 0.685 for one-year, 0.744 for two-year, and 0.777 for three-years (Fig 3F). This means the nomograms have a good ability to accurately predict outcomes or risks. Furthermore, to explore whether prognosis was associated with clinical features, we stratified the patients into subgroups based on age or gender. The high-risk group (age < 59) years had a worse prognosis, while similar results were observed for female and male patients (Fig 3G–J). Additionally, the PFS showed statistically significant differences between the high- and low-risk groups (p <  0.05, Fig 3K). These results further confirm the predictive ability of our risk model.

### Association between CRLs and immune cells

**Fig 4:**
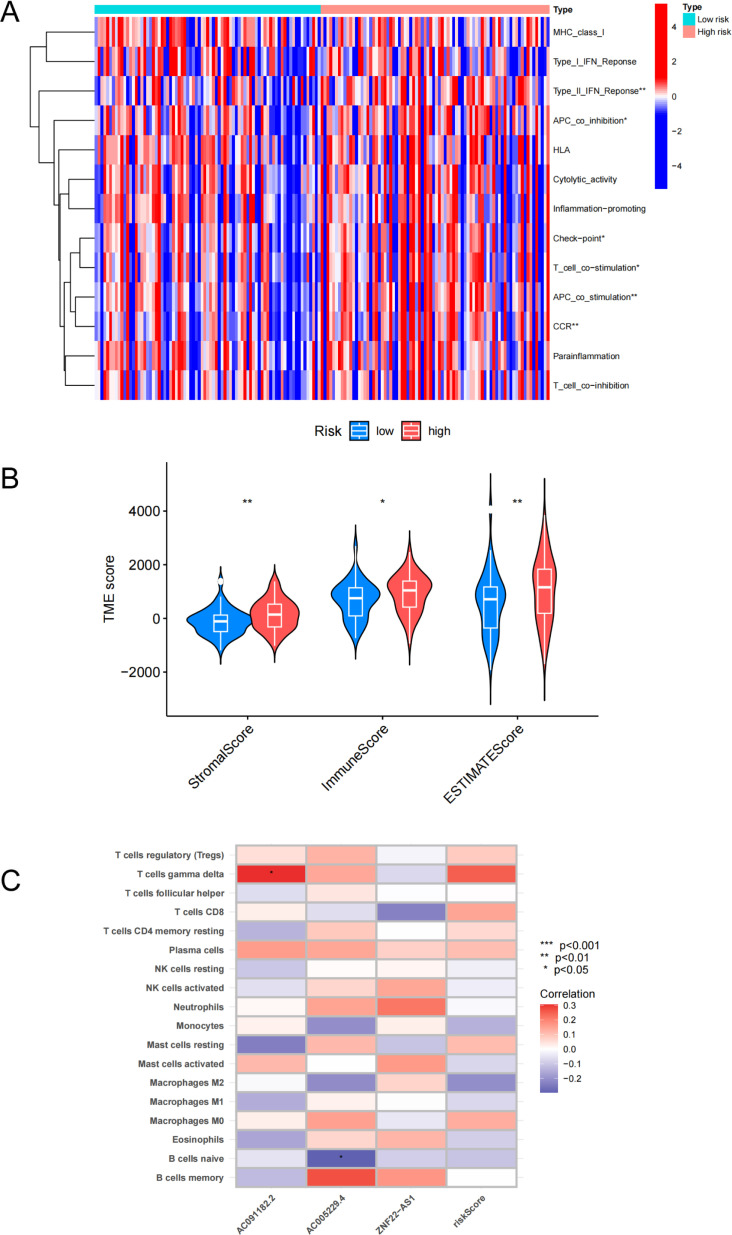
Relationship of risk score with immune status. (A) Analysis of correlation and difference between high- and low-risk groups and immune pathways. (B) The violin diagram shows the three kinds of TME scores in the high-risk group and low-risk groups. * p <  0.05, **p <  0.01, ***p < 0.001. (C) Correlation heatmap of Spearman correlation analyses between 3 model-related CRLs and the contents of immune cells.

For additional insight into the mechanisms of GBM, we analyzed intratumoral immune cell infiltration in the high- and low-risk groups. The ssGSEA methodology was utilized to compare the immune cell infiltration in low- and high-risk groups from the clinical samples (n = 159). Correlation analysis between immune cell populations indicated that there were significant differences in type-II IFN response, APC co-inhibition, T-cell co-stimulation and APC co-stimulation between the high- and low-risk groups (Fig 4A). The stromal score, immune score and ESTIMATE score of low-risk GBM samples were considerably lower than those of high-risk GBM samples (Fig 4B). Furthermore, there was a strong association between the types of immune cells and the expression levels of the 3 CRLs (Fig 4C). T cells and AC091182.2 had a significant positive correlation (p < 0.05), while there was a strong negative correlation between naïve B cells and AC005229.4 (p < 0.05).

### The high-risk CRLs and CRGs are highly expressed in human GBM cell lines and the G422-GBM mouse model

**Fig 5:**
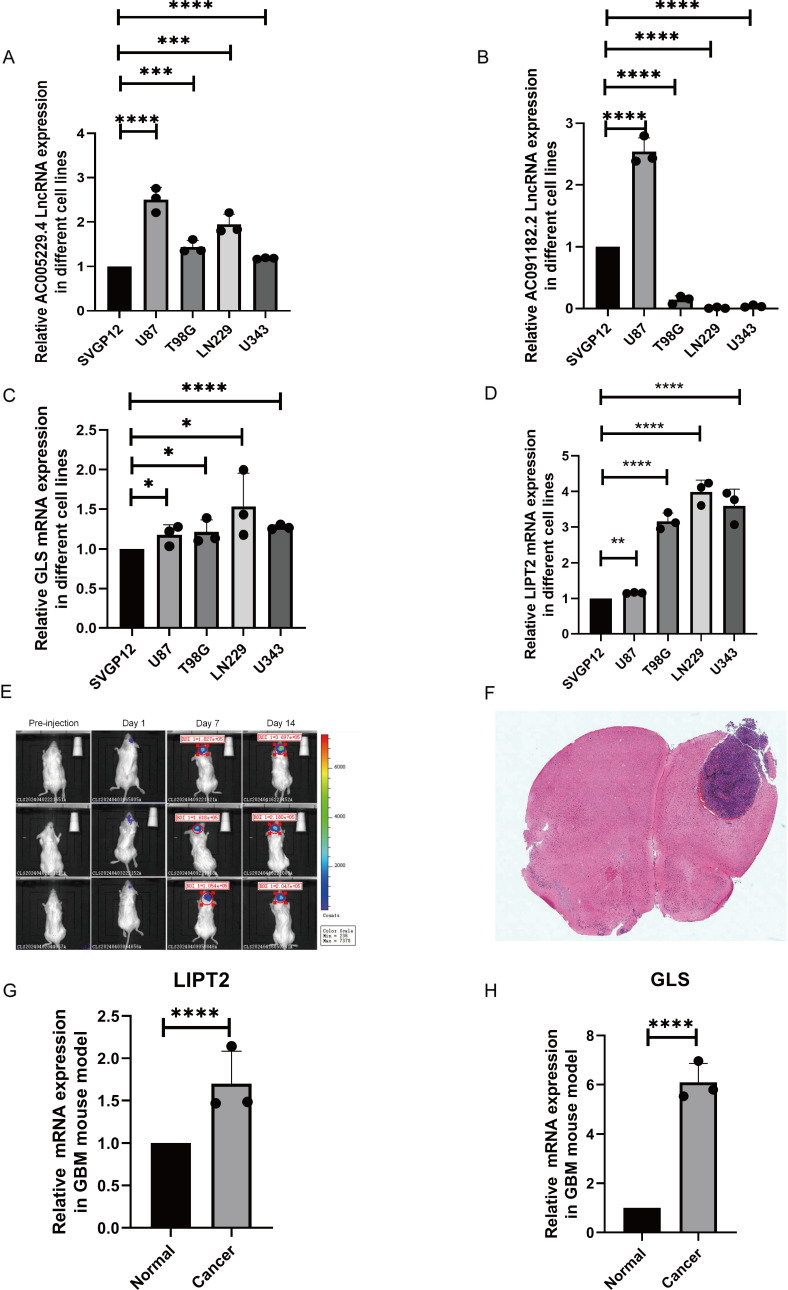
Quantitative real-time PCR analysis of high-risk CRLs and co-expressed CRGs in GBM human cell lines and the GBM mouse model. (A–D) Relative lncRNA and mRNA expression in normal astrocyte (SVGP12) and GBM cell lines (U87, T98G, U343, LN229): (A) AC005229.4; (B) AC091182.2; (C) LIPT2; (D) GLS. (E) Tumor volumes were analyzed by bioluminescence imaging. (F) Hematoxylin and eosin staining of a representative GBM mouse brain (20×). (G-H) Relative mRNA expression in mouse GBM and normal brain tissues: (G) LIPT2; (H) GLS. * p <  0.05, **p <  0.01, ***p <  0.001, ****p <  0.0001.

To verify the expression of the high-risk CRLs and their CRGs, we performed RT-qPCR of GBM and normal astrocyte cells. The expression of AC005229.4 was significantly higher in all GBM cell lines tested than in SVGP12 astrocytes (Fig 5A). Furthermore, the expression of AC091182.2 was higher in U87 cells than in SVGP12, but was lower all in other GBM cell lines (Fig 5B). The mRNA expression of *GLS* and *LIPT2* was significantly higher in all GBM cells than in SVGP12 cells (Fig 5C–D).

We sought to further evaluate the in vivo expression of these CLRs and CRGs. Thus, to simulate the complex brain microenvironment, we constructed an orthotopic G422-GFP-LUC GBM mouse model. Because AC005229.4 and AC091182.2 were of human origin and did not correspond to any mouse lncRNAs in public online databases, only the expression of *LIPT2* and *GLS* could be validated in the G422-GFP-LUC GBM mouse model. The tumor implantation status was verified using bioluminescence imaging (Fig 5E), and the tumor extent was determined by HE staining (Fig 5F). The results showed that the expression of both *LIPT2* and *GLS* was elevated in the mouse GBM tissues compared with the normal mouse brain tissues (Fig 5G–H), which further confirms the in vitro data and is consistent with the results of the bioinformatics analysis.

### The expression of AC091182.2, AC005229.4, *LIPT2* and *GLS* is decreased by GBM cuproptosis

 To verify the relevance of the selected factors to cuproptosis, we established a GBM cuproptosis cell model. This model for constructing cellular cuproptosis has been validated in the literature [17,18]. Moreover, it has been shown to cause only cuproptosis and no other forms of cell death. As in previous studies [17–19,27], a concentration gradient and time gradient of Elesclomol and CuCl_2_ were applied (S1 Fig). Finally, 10 μM CuCl_2_ and 40 nM Elesclomol were selected as the optimal administration concentrations, and 2 hours was selected as the optimal administration time.

**Fig 6:**
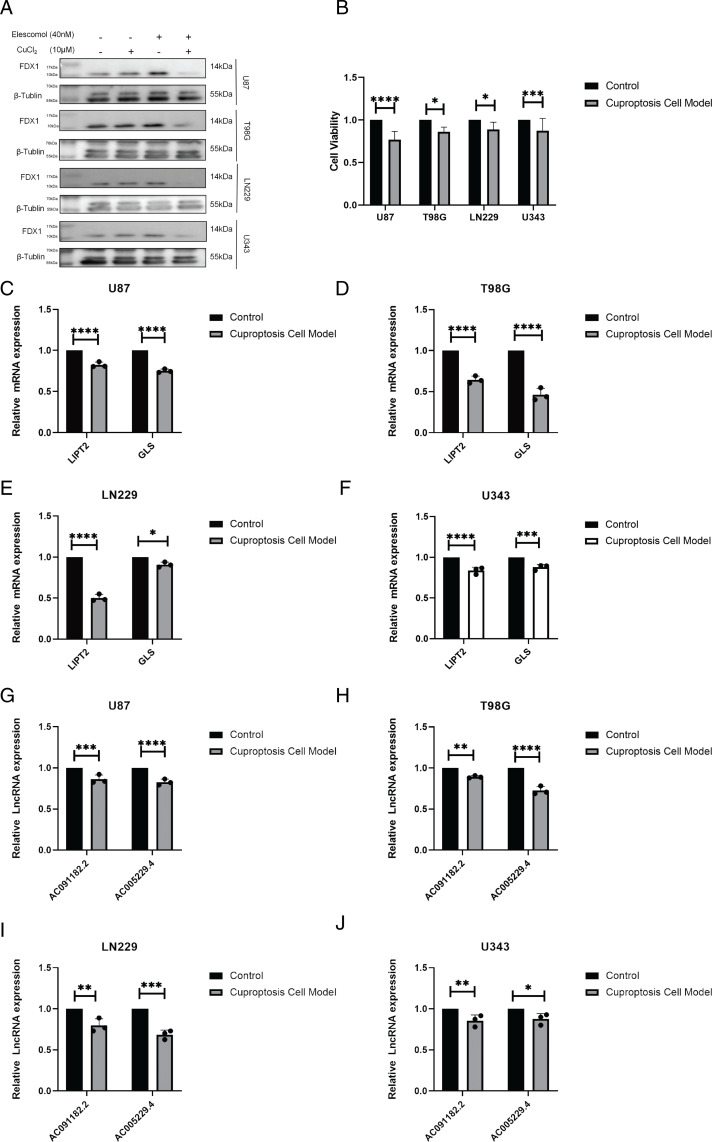
Expression of high-risk CRLs and co-expression CRGs in the GBM cuproptosis model. (A) Western blot analysis of GBM cell lines treated with 10 μM CuCl_2_ and 40 nM Elesclomol for 2 h. (B) Cell viability of the GBM cuproptosis model as evaluated by CCK-8 assay. (C-J) Quantitative real-time PCR analysis of AC005229.4, AC091182.2, LIPT2 and GLS in the GBM cuproptosis model. * p <  0.05, **p <  0.01, ***p <  0.001. ns, no significance.

According to the available literature, the internal Fe-S cluster protein FDX1 is a recognized marker for cuproptosis [17,18]. Therefore, we performed western blot experiments to detect the protein expression changes of FDX1 in the cuproptosis GBM cell model. As expected, FDX1 protein levels were decreased in the CuCl_2_/ Elesclomol co-administration group compared to the control and single-administration groups (Fig 6A). Furthermore, the cell viability was decreased in the cuproptosis group as compared to the control group as evaluated by CCK-8 assay (Fig 6B). These results confirm the successful construction of the GBM cuproptosis model.

Next, we assessed the expression of the high-risk CRLs and CRGs in the GBM cuproptosis cell model. The mRNA levels of AC091182.2, AC005229.4, *LIPT2* and *GLS* were all decreased in the cuproptosis group as compared to the control group (Fig 6C–J). These results suggest that AC091182.2, AC005229.4, *LIPT2* and *GLS* are associated with GBM cuproptosis.

### AC091182.2, AC005229.4, *LIPT2* and *GLS* may serve as GBM cuproptosis-related drug indicators

**Fig 7:**
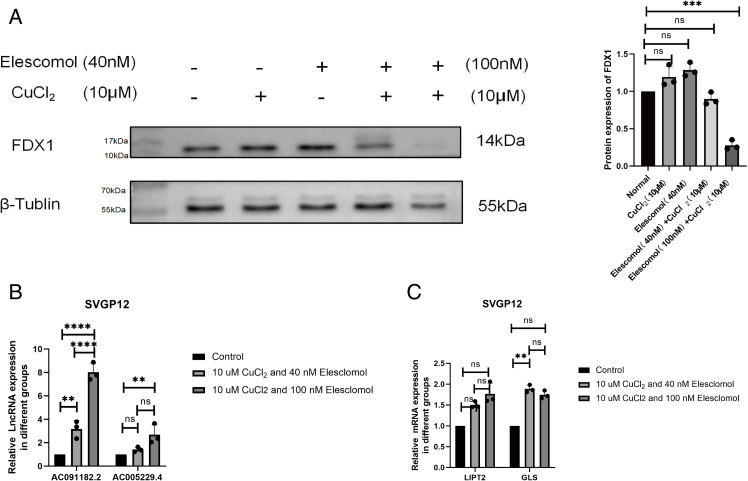
Drug concentrations for constructing a normal astrocyte cuproptosis model and expression of high-risk CRLs and co-expression CRGs in the human astrocyte cuproptosis model. (A) Western blot analysis of svgp12 human astrocyte cells treated with 10 μM CuCl2 and/or 40–100 nM Elesclomol for 2 h. (B–E) Quantitative real-time PCR analysis of AC005229.4, AC091182.2, LIPT2 and GLS in the normal astrocyte cuproptosis model (10 μM CuCl2 and 100 nM Elesclomol). * p <  0.05, **p <  0.01, ***p <  0.001. ns, not significant.

To evaluate the effects of cuproptosis drugs in normal astrocytes, we constructed a svgp12 cell cuproptosis model. In the svgp12 cells, effects on FDX1 expression were not detected at the same drug concentrations used for the GBM cells (10 μM CuCl_2_ and 40 nM Elesclomol), though higher drug concentrations (10 μM CuCl_2_ and 100 nM Elesclomol) effectively induced cuproptosis, as indicated by decreased FDX1 expression (Fig 7A). These results suggest that GBM cell lines may be more sensitive than normal astrocytes to drug-induced cuproptosis.

After treatment with either low or high drug doses, the expression of AC091182.2, AC005229.4, *LIPT2* and *GLS* of was elevated (Fig 7B–C). This trend is the opposite of that observed for GBM cells (Fig 6C–J), indicating that AC091182.2, AC005229.4, *LIPT2* and *GLS* may serve as GBM-specific indicators of cuproptosis in GBM.

## Discussion

Despite recent improvements in surgical techniques and chemotherapeutic agents, the prognosis for GBM patients remains poor, and resistance to conventional chemotherapeutic agents is inevitable [26,28]. RCD has been demonstrated to be crucial in the prediction of cancer patients’ outcomes, cancer progression/metastasis, and cancer immunosurveillance, as well as in combating chemotherapy resistance [29]. Furthermore, the combination of conventional therapies and RCD modulators has great potential for cancer treatment [30]. Cuproptosis is a more recently characterized form of RCD that is largely dependent on Cu induction and is associated with mitochondrial metabolism, tumor proliferation, metastasis and drug resistance [29,31,32].

In this study, we sought to explore which cuproptosis-related genes and lncRNAs are associated with the development of GBM. Key molecules in RCD have been shown to be regulated by lncRNAs, influencing tumor progression, migration, invasion and resistance to chemotherapeutic agents [33–35]. Additionally, recent studies have used online databases to construct clinical prognostic models of cuproptosis hub genes or lncRNAs associated with prognostic significance and immune characteristics for gliomas [20,36–39]. However, this is the first bioinformatic analysis to construct a cuproptosis-associated lncRNA predictive model specifically for GBM, the highest-grade glioma.

To identify lncRNAs associated with GBM, we bioinformatically analyzed the GBM dataset from TCGA and constructed a GBM cuproptosis-related lncRNA prediction model. High-risk CRLs (AC091182.2, AC005229.4) and CRGs (*LIPT2*, *GLS*) were identified though the model. AC005229.4 has been previously associated with prognosis of endometrial cancer, bladder cancer and hepatocellular carcinoma [40–42]. However, to our knowledge, this is the first study to associate the lncRNA AC091182.2 with cancer. Previously, *LIPT2* expression has been associated with bad prognosis in glioma [43], and GLS is recognized as a factor associated with malignant proliferation and invasion in gliomas [44–46]. Thus, the prior identification of AC005229.4, *LIPT2* and *GLS* as having a role in the advancement of gliomas and other types of cancer provides support for our findings. Notably, cuproptosis has been demonstrated in a variety of malignancies (e.g., breast [47], thyroid [48], leukemia [49] and oral cancer [50]), and changes in copper homeostasis has been shown to affect tumor progression or aggressiveness, as well as the development of resistance to treatment [51]. Therefore, the findings from our study provide insights into the role of cuproptosis in GBM and may have relevance to cancers other than GBM.

For experimental validation, we examined the expression of the high-risk CRLs and CRGs in GBM cells and a GBM mouse model. Furthermore, we developed a cuproptosis cell model using GBM cells and astrocyte to verify associations with cuproptosis. According to the literature, the internal Fe-S cluster protein FDX1 is a recognized marker for cuproptosis [17,18]. The results revealed that these factors are highly expressed in GBM, and their expression is decreased when cuproptosis is induced in GBM cells. The doses of cuproptosis-inducing agents that mediated cuproptosis, as indicated by decreased FDX1 expression, were higher for normal astrocytes than for GBM cells, suggesting that GBM cells may be more sensitive than astrocytes to cuproptosis-inducing drugs. Moreover, the expression of the high-risk CRLs and CRGs was elevated in both lower and higher drug doses in normal astrocytes, which is opposite to the pattern observed for GBM cells. Therefore, the identified CRLs and CRGs may serve as a marker for the detection of cuproptosis in GBM, as well as an indicator for preventing adverse effects of cuproptosis-inducing drug overdose on normal brain cells.

Though we focused on cuproptosis in this study, our study also highlighted the contributions of related processes, such as intratumoral immune cell infiltration. The characteristics of immune infiltrating cells are considered to be an additional prognostic factor in glioma [52,53]. In previous studies, different forms of RCD may alter the tumor microenvironment (TME) by releasing pathogens or damage-associated molecular patterns, which can influence the efficacy of anti-cancer therapy [54–56]. Because copper is known to influence immune response, exploring TME cell infiltration could enhance our comprehension of the GBM tumor response to anti-tumor medication [57,58]. Our results show that gamma delta T cells had a positive association with AC091182.2, whereas naïve B cells exhibited a negative correlation with AC005229.4. The importance of gamma delta cells in GBM has been established [59,60]. Thus, this raising the possibility of co-regulation between cuproptosis and immune pathways that impact GBM outcome.

While our research advances the understanding of GBM by providing a novel prognostic model for GBM using CRLs and CRGs associated with cuproptosis to facilitate personalized prediction of prognosis, treatment and recurrence, the study has some limitations. First, the TCGA database has a limited number of GBM samples with prognostic information, thus restraining our capacity to validate the predictive signature’s utility. Furthermore, additional testing will be necessary to clarify the distinct functions of the CRLs and CRGs in GBM cuproptosis-related processes, including immune infiltration, tumor invasion, metastasis and drug resistance. Future research to address these limitations would help to extend our findings.

## Conclusions

In conclusion, we have constructed a predictive model based on CRLs and CRGs and have demonstrated that the identified CRLs and CRGs may serve as specific biomarkers for GBM and indicators for cuproptosis-related drugs. The results of this study may be useful in informing future investigations about the underlying mechanisms of cuproptosis, as well as strategies for the development of new potential treatment options for GBM. Therefore, our results further add to the understanding of cuproptosis in GBM and provide theoretical support for future treatment.

## Supporting information

S1 Fig Status of GBM cell lines at different drug concentrations and times. (PDF)

S2 Fig The session information of R in this study. (PDF)

S1 Table The clinical characteristics of patients in the TCGA database. (PDF)

S2 Table Sequences of primers used in this research. (PDF)

S3 Table Multivariate Cox regression analysis. (PDF)

S1 Raw Images Western blotting of the cuproptosis cell model for three consecutive independent replicate experiments. (PDF)

## References

[1] Weller M, Wick W, Aldape K, Brada M, Berger M, Pfister SM, et al. Glioma. Nat Rev Dis Primers. 2015;1:15017. doi: 10.1038/nrdp.2015.17 27188790

[2] Louis DN, Perry A, Wesseling P, Brat DJ, Cree IA, Figarella-Branger D, et al. The 2021 WHO classification of tumors of the central nervous system: a summary. Neuro Oncol. 2021;23(8):1231–51. doi: 10.1093/neuonc/noab106 34185076 PMC8328013

[3] Samioti SE, Benos LT, Sarris IE. Effect of fractal-shaped outer boundary of glioblastoma multiforme on drug delivery. Comput Methods Programs Biomed. 2019;178:191–9. doi: 10.1016/j.cmpb.2019.06.031 31416549

[4] Polivka J Jr, Polivka J, Holubec L, Kubikova T, Priban V, Hes O, et al. Advances in experimental targeted therapy and immunotherapy for patients with glioblastoma multiforme. Anticancer Res. 2017;37(1):21–33. doi: 10.21873/anticanres.11285 28011470

[5] Mariathasan S, Turley SJ, Nickles D, Castiglioni A, Yuen K, Wang Y, et al. TGFβ attenuates tumour response to PD-L1 blockade by contributing to exclusion of T cells. Nature. 2018;554(7693):544–8. doi: 10.1038/nature25501 29443960 PMC6028240

[6] Molinaro AM, Wrensch MR, Jenkins RB, Eckel-Passow JE. Statistical considerations on prognostic models for glioma. Neuro Oncol. 2016;18(5):609–23. doi: 10.1093/neuonc/nov255 26657835 PMC4827041

[7] Bush AI. Metals and neuroscience. Curr Opin Chem Biol. 2000;4(2):184–91. doi: 10.1016/s1367-5931(99)00073-3 10742195

[8] Thiele DJ. Integrating trace element metabolism from the cell to the whole organism. J Nutr. 2003;133(5 Suppl 1):1579S–80S. doi: 10.1093/jn/133.5.1579S 12730470

[9] Peña MM, Lee J, Thiele DJ. A delicate balance: homeostatic control of copper uptake and distribution. J Nutr. 1999;129(7):1251–60. doi: 10.1093/jn/129.7.1251 10395584

[10] Zheng W, Monnot AD. Regulation of brain iron and copper homeostasis by brain barrier systems: implication in neurodegenerative diseases. Pharmacol Ther. 2012;133(2):177–88. doi: 10.1016/j.pharmthera.2011.10.006 22115751 PMC3268876

[11] Kardos J, Héja L, Simon Á, Jablonkai I, Kovács R, Jemnitz K. Copper signalling: causes and consequences. Cell Commun Signal. 2018;16(1):71. doi: 10.1186/s12964-018-0277-3 30348177 PMC6198518

[12] Stuerenburg HJ. CSF copper concentrations, blood-brain barrier function, and coeruloplasmin synthesis during the treatment of Wilson’s disease. J Neural Transm (Vienna). 2000;107(3):321–9. doi: 10.1007/s007020050026 10821440

[13] An Y, Li S, Huang X, Chen X, Shan H, Zhang M. The role of copper homeostasis in brain disease. Int J Mol Sci. 2022;23(22):13850. doi: 10.3390/ijms232213850 36430330 PMC9698384

[14] Gromadzka G, Tarnacka B, Flaga A, Adamczyk A. Copper dyshomeostasis in neurodegenerative diseases-therapeutic implications. Int J Mol Sci. 2020;21(23):9259. doi: 10.3390/ijms21239259 33291628 PMC7730516

[15] Porporato PE, Filigheddu N, Pedro JMB-S, Kroemer G, Galluzzi L. Mitochondrial metabolism and cancer. Cell Res. 2018;28(3):265–80. doi: 10.1038/cr.2017.155 29219147 PMC5835768

[16] Sriskanthadevan S, Jeyaraju DV, Chung TE, Prabha S, Xu W, Skrtic M, et al. AML cells have low spare reserve capacity in their respiratory chain that renders them susceptible to oxidative metabolic stress. Blood. 2015;125(13):2120–30. doi: 10.1182/blood-2014-08-594408 25631767 PMC4375109

[17] Tsvetkov P, Coy S, Petrova B, Dreishpoon M, Verma A, Abdusamad M, et al. Copper induces cell death by targeting lipoylated TCA cycle proteins. Science. 2022;375(6586):1254–61. doi: 10.1126/science.abf0529 35298263 PMC9273333

[18] Tsvetkov P, Detappe A, Cai K, Keys HR, Brune Z, Ying W, et al. Mitochondrial metabolism promotes adaptation to proteotoxic stress. Nat Chem Biol. 2019;15(7):681–9. doi: 10.1038/s41589-019-0291-9 31133756 PMC8183600

[19] Buccarelli M, D’Alessandris QG, Matarrese P, Mollinari C, Signore M, Cappannini A, et al. Elesclomol-induced increase of mitochondrial reactive oxygen species impairs glioblastoma stem-like cell survival and tumor growth. J Exp Clin Cancer Res. 2021;40(1):228. doi: 10.1186/s13046-021-02031-4 34253243 PMC8273992

[20] Zhang B, Xie L, Liu J, Liu A, He M. Construction and validation of a cuproptosis-related prognostic model for glioblastoma. Front Immunol. 2023;14:1082974. doi: 10.3389/fimmu.2023.1082974 36814929 PMC9939522

[21] Zhou Y, Xiao D, Jiang X, Nie C. EREG is the core onco-immunological biomarker of cuproptosis and mediates the cross-talk between VEGF and CD99 signaling in glioblastoma. J Transl Med. 2023;21(1):28. doi: 10.1186/s12967-023-03883-4 36647156 PMC9843967

[22] Lu H, Zhou L, Zhang B, Xie Y, Yang H, Wang Z. Corrigendum: cuproptosis key gene FDX1 is a prognostic biomarker and associated with immune infiltration in glioma. Front Med (Lausanne). 2023;10:1244638. doi: 10.3389/fmed.2023.1244638 37492249 PMC10364843

[23] Li D, Wang Y, Dong C, Chen T, Dong A, Ren J, et al. CST1 inhibits ferroptosis and promotes gastric cancer metastasis by regulating GPX4 protein stability via OTUB1. Oncogene. 2023;42(2):83–98. doi: 10.1038/s41388-022-02537-x 36369321 PMC9816059

[24] Gai C, Liu C, Wu X, Yu M, Zheng J, Zhang W, et al. MT1DP loaded by folate-modified liposomes sensitizes erastin-induced ferroptosis via regulating miR-365a-3p/NRF2 axis in non-small cell lung cancer cells. Cell Death Dis. 2020;11(9):751. doi: 10.1038/s41419-020-02939-3 32929075 PMC7490417

[25] Hänzelmann S, Castelo R, Guinney J. GSVA: gene set variation analysis for microarray and RNA-seq data. BMC Bioinformatics. 2013;14:7. doi: 10.1186/1471-2105-14-7 23323831 PMC3618321

[26] Newman AM, Liu CL, Green MR, Gentles AJ, Feng W, Xu Y, et al. Robust enumeration of cell subsets from tissue expression profiles. Nat Methods. 2015;12(5):453–7. doi: 10.1038/nmeth.3337 25822800 PMC4739640

[27] Merker K, Hapke D, Reckzeh K, Schmidt H, Lochs H, Grune T. Copper related toxic effects on cellular protein metabolism in human astrocytes. Biofactors. 2005;24(1–4):255–61. doi: 10.1002/biof.5520240130 16403986

[28] Davis ME. Epidemiology and Overview of Gliomas. Semin Oncol Nurs. 2018;34(5):420–9. doi: 10.1016/j.soncn.2018.10.001 30392758

[29] Tong X, Tang R, Xiao M, Xu J, Wang W, Zhang B, et al. Targeting cell death pathways for cancer therapy: recent developments in necroptosis, pyroptosis, ferroptosis, and cuproptosis research. J Hematol Oncol. 2022;15(1):174. doi: 10.1186/s13045-022-01392-3 36482419 PMC9733270

[30] Gong Y, Fan Z, Luo G, Yang C, Huang Q, Fan K, et al. The role of necroptosis in cancer biology and therapy. Mol Cancer. 2019;18(1):100. doi: 10.1186/s12943-019-1029-8 31122251 PMC6532150

[31] Tang D, Chen X, Kroemer G. Cuproptosis: a copper-triggered modality of mitochondrial cell death. Cell Res. 2022;32(5):417–8. doi: 10.1038/s41422-022-00653-7 35354936 PMC9061796

[32] Yu D, Liu C, Guo L. Mitochondrial metabolism and cancer metastasis. Ann Transl Med. 2020;8(14):904. doi: 10.21037/atm.2020.03.42 32793748 PMC7396750

[33] Shuai Y, Ma Z, Liu W, Yu T, Yan C, Jiang H, et al. TEAD4 modulated LncRNA MNX1-AS1 contributes to gastric cancer progression partly through suppressing BTG2 and activating BCL2. Mol Cancer. 2020;19(1):6. doi: 10.1186/s12943-019-1104-1 31924214 PMC6953272

[34] Liu S-Y, Zhao Z-Y, Qiao Z, Li S-M, Zhang W-N. LncRNA PCAT1 Interacts with DKC1 to regulate proliferation, invasion and apoptosis in NSCLC cells via the VEGF/AKT/Bcl2/Caspase9 pathway. Cell Transplant. 2021;30:963689720986071. doi: 10.1177/0963689720986071 33461333 PMC7818005

[35] Liu T, Hu J, Han B, Tan S, Jia W, Xin Y. A positive feedback loop of lncRNA-RMRP/ZNRF3 axis and Wnt/β-catenin signaling regulates the progression and temozolomide resistance in glioma. Cell Death Dis. 2021;12(11):952. doi: 10.1038/s41419-021-04245-y 34657141 PMC8520527

[36] Wu Z, Li W, Zhu H, Li X, Zhou Y, Chen Q, et al. Identification of cuproptosis-related subtypes and the development of a prognostic model in glioma. Front Genet. 2023;14:1124439. doi: 10.3389/fgene.2023.1124439 36936439 PMC10014798

[37] Wang W, Lu Z, Wang M, Liu Z, Wu B, Yang C, et al. The cuproptosis-related signature associated with the tumor environment and prognosis of patients with glioma. Front Immunol. 2022;13:998236. doi: 10.3389/fimmu.2022.998236 36110851 PMC9468372

[38] Chen S, Zhang S, Yuan Y, Wang Z, Li J, Li T, et al. Prognostic value of cuproptosis-related genes signature and its impact on the reshaped immune microenvironment of glioma. Front Pharmacol. 2022;13:1016520. doi: 10.3389/fphar.2022.1016520 36267281 PMC9576857

[39] Huang W, Wu Y, Zhu J, Luo N, Wang C, Liu S, et al. Pan-cancer integrated bioinformatics analysis reveals cuproptosis related gene FDX1 is a potential prognostic and immunotherapeutic biomarker for lower-grade gliomas. Front Mol Biosci. 2023;10 963639. doi: 10.3389/fmolb.2023.963639 36825202 PMC9941349

[40] Wang X, Dai C, Ye M, Wang J, Lin W, Li R. Prognostic value of an autophagy-related long-noncoding-RNA signature for endometrial cancer. Aging (Albany NY). 2021;13(4):5104–19. doi: 10.18632/aging.202431 33534780 PMC7950257

[41] Wan J, Guo C, Fang H, Xu Z, Hu Y, Luo Y. Autophagy-related long non-coding RNA is a prognostic indicator for bladder cancer. Front Oncol. 2021;11:647236. doi: 10.3389/fonc.2021.647236 33869042 PMC8049181

[42] Jia Y, Chen Y, Liu J. Prognosis-predictive signature and nomogram based on autophagy-related long non-coding RNAs for hepatocellular carcinoma. Front Genet. 2020;11:608668. doi: 10.3389/fgene.2020.608668 33424932 PMC7793718

[43] Wang W, Li S, Huang Y, Guo J, Sun L, Sun G. Comprehensive analysis of the potential biological significance of cuproptosis-related gene LIPT2 in pan-cancer prognosis and immunotherapy. Sci Rep. 2023;13(1):22910. doi: 10.1038/s41598-023-50039-x 38129565 PMC10739704

[44] Martín-Rufián M, Nascimento-Gomes R, Higuero A, Crisma AR, Campos-Sandoval JA, Gómez-García MC, et al. Both GLS silencing and GLS2 overexpression synergize with oxidative stress against proliferation of glioma cells. J Mol Med (Berl). 2014;92(3):277–90. doi: 10.1007/s00109-013-1105-2 24276018 PMC4327995

[45] Yamashita AS, da Costa Rosa M, Stumpo V, Rais R, Slusher BS, Riggins GJ. The glutamine antagonist prodrug JHU-083 slows malignant glioma growth and disrupts mTOR signaling. Neurooncol Adv. 2020;3(1):vdaa149. doi: 10.1093/noajnl/vdaa149 33681764 PMC7920530

[46] Panosyan EH, Lasky JL, Lin HJ, Lai A, Hai Y, Guo X, et al. Clinical aggressiveness of malignant gliomas is linked to augmented metabolism of amino acids. J Neurooncol. 2016;128(1):57–66. doi: 10.1007/s11060-016-2073-5 26922345 PMC5373108

[47] Pavithra V, Sathisha TG, Kasturi K, Mallika DS, Amos SJ, Ragunatha S. Serum levels of metal ions in female patients with breast cancer. J Clin Diagn Res. 2015;9(1):BC25–c27. doi: 10.7860/JCDR/2015/11627.5476 25737978 PMC4347069

[48] Baltaci AK, Dundar TK, Aksoy F, Mogulkoc R. Changes in the serum levels of trace elements before and after the operation in thyroid cancer patients. Biol Trace Elem Res. 2017;175(1):57–64. doi: 10.1007/s12011-016-0768-2 27263537

[49] Zuo XL, Chen JM, Zhou X, Li XZ, Mei GY. Levels of selenium, zinc, copper, and antioxidant enzyme activity in patients with leukemia. Biol Trace Elem Res. 2006;114(1–3):41–53. doi: 10.1385/BTER:114:1:41 17205986

[50] Baharvand M, Manifar S, Akkafan R, Mortazavi H, Sabour S. Serum levels of ferritin, copper, and zinc in patients with oral cancer. Biomed J. 2014;37(5):331–6. doi: 10.4103/2319-4170.132888 25179706

[51] Lelièvre P, Sancey L, Coll J-L, Deniaud A, Busser B. The multifaceted roles of copper in cancer: a trace metal element with dysregulated metabolism, but also a target or a bullet for therapy. Cancers (Basel). 2020;12(12):3594. doi: 10.3390/cancers12123594 33271772 PMC7760327

[52] Zhang N, Zhang H, Wu W, Zhou R, Li S, Wang Z, et al. Machine learning-based identification of tumor-infiltrating immune cell-associated lncRNAs for improving outcomes and immunotherapy responses in patients with low-grade glioma. Theranostics. 2022;12(13):5931–48. doi: 10.7150/thno.74281 35966587 PMC9373811

[53] Kim AR, Choi KS, Kim M-S, Kim K-M, Kang H, Kim S, et al. Absolute quantification of tumor-infiltrating immune cells in high-grade glioma identifies prognostic and radiomics values. Cancer Immunol Immunother. 2021;70(7):1995–2008. doi: 10.1007/s00262-020-02836-w 33416947 PMC10991432

[54] Wang X, Wu S, Liu F, Ke D, Wang X, Pan D, et al. An immunogenic cell death-related classification predicts prognosis and response to immunotherapy in head and neck squamous cell carcinoma. Front Immunol. 2021;12:781466. doi: 10.3389/fimmu.2021.781466 34868055 PMC8640500

[55] Chen X, Zeh HJ, Kang R, Kroemer G, Tang D. Cell death in pancreatic cancer: from pathogenesis to therapy. Nat Rev Gastroenterol Hepatol. 2021;18(11):804–23. doi: 10.1038/s41575-021-00486-6 34331036

[56] Wang H, Liu M, Zeng X, Zheng Y, Wang Y, Zhou Y. Cell death affecting the progression of gastric cancer. Cell Death Discov. 2022;8(1):377. doi: 10.1038/s41420-022-01161-8 36038533 PMC9424204

[57] Percival SS. Copper and immunity. Am J Clin Nutr. 1998;67(5 Suppl):1064S–8S. doi: 10.1093/ajcn/67.5.1064S 9587153

[58] Culbertson EM, Culotta VC. Copper in infectious disease: using both sides of the penny. Semin Cell Dev Biol. 2021;115:19–26. doi: 10.1016/j.semcdb.2020.12.003 33423931

[59] Bryant NL, Suarez-Cuervo C, Gillespie GY, Markert JM, Nabors LB, Meleth S, et al. Characterization and immunotherapeutic potential of gammadelta T-cells in patients with glioblastoma. Neuro Oncol. 2009;11(4):357–67. doi: 10.1215/15228517-2008-111 19211933 PMC2743216

[60] Han S, Feng S, Ren M, Ma E, Wang X, Xu L, et al. Glioma cell-derived placental growth factor induces regulatory B cells. Int J Biochem Cell Biol. 2014;57:63–8. doi: 10.1016/j.biocel.2014.10.005 25450457

