# Supplementary material for: Cuproptosis-related lncRNAs and genes: Potential markers for glioblastoma prognosis and treatment
Source: PLoS One. 2025 Feb 6;20(2):e0315927. doi: 10.1371/journal.pone.0315927 (PMC11801720; doi:10.1371/journal.pone.0315927)
Supplement: S1 Fig — (PDF) [file pone.0315927.s001.pdf]

Supplementary Figure S1:
Status of GBM cell lines at different drug concentrations and times.

The optimal dosing time for all GBM cell lines was 2 hours, and the optimal dosing concentrations were 10 uM $CuCl_2$ and 40 nM Elesclomol.

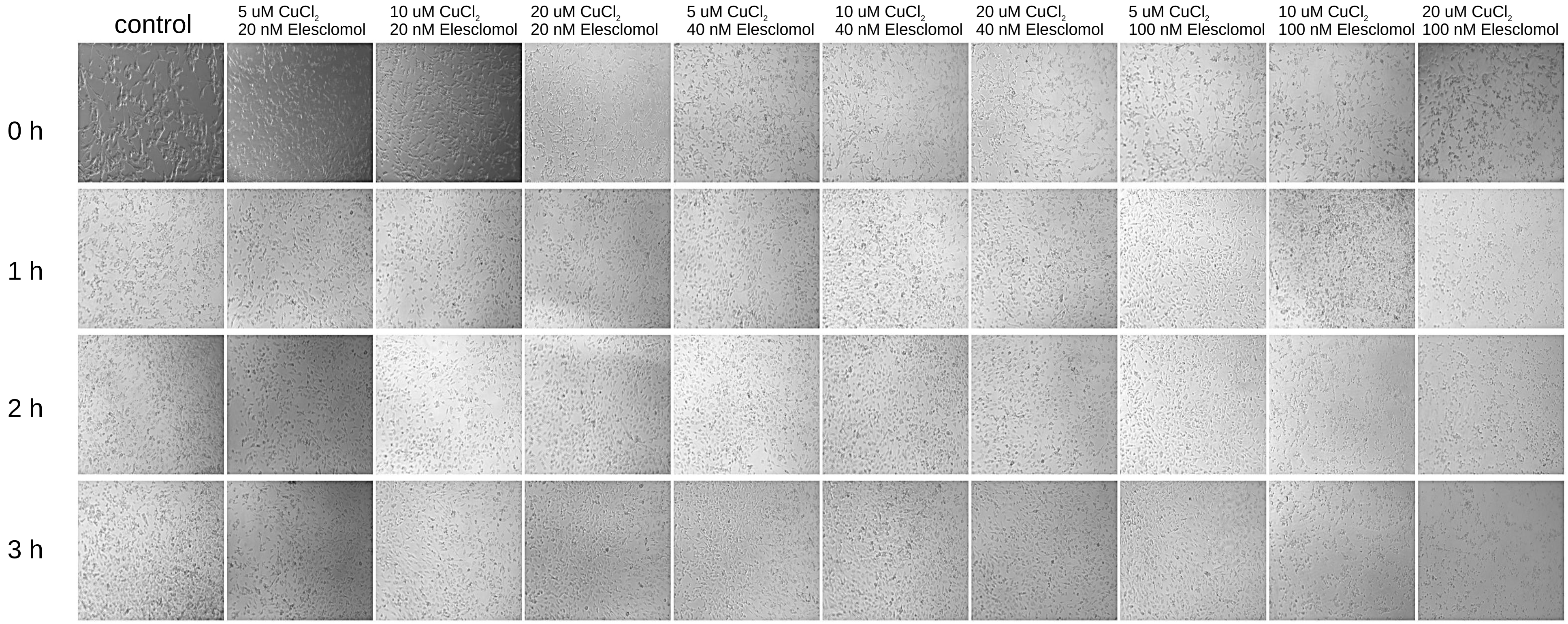

Figure 1.Status of U87 at different drug concentrations and times.

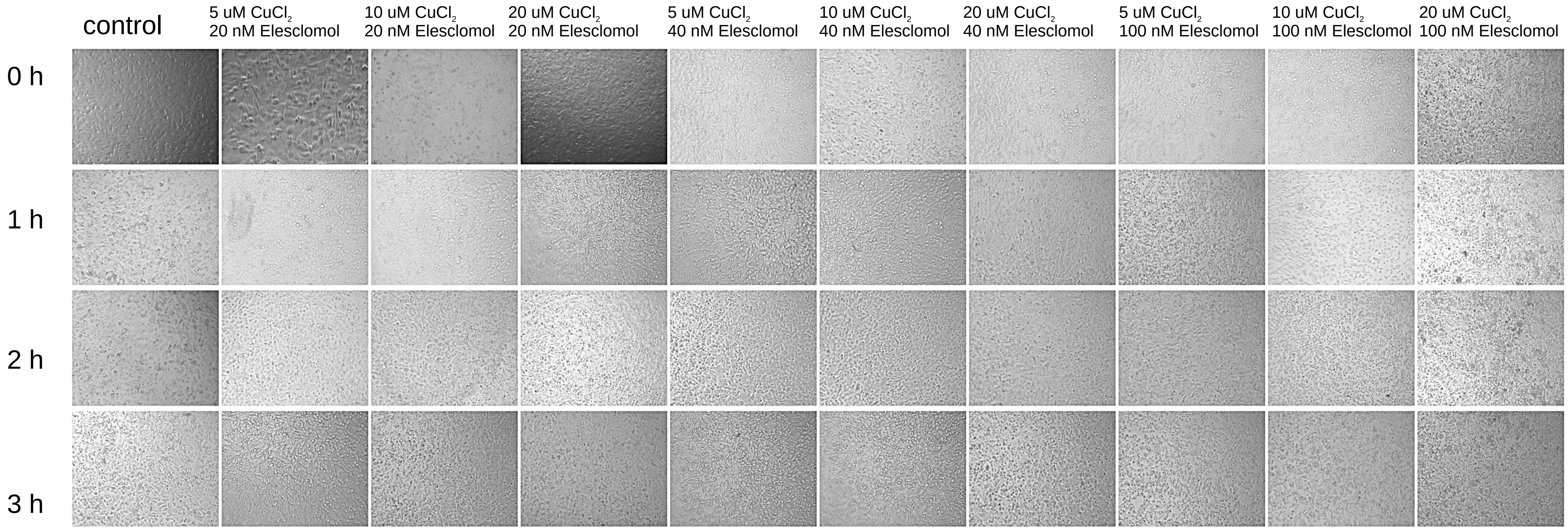

Figure 2.Status of T989G at different drug concentrations and times.

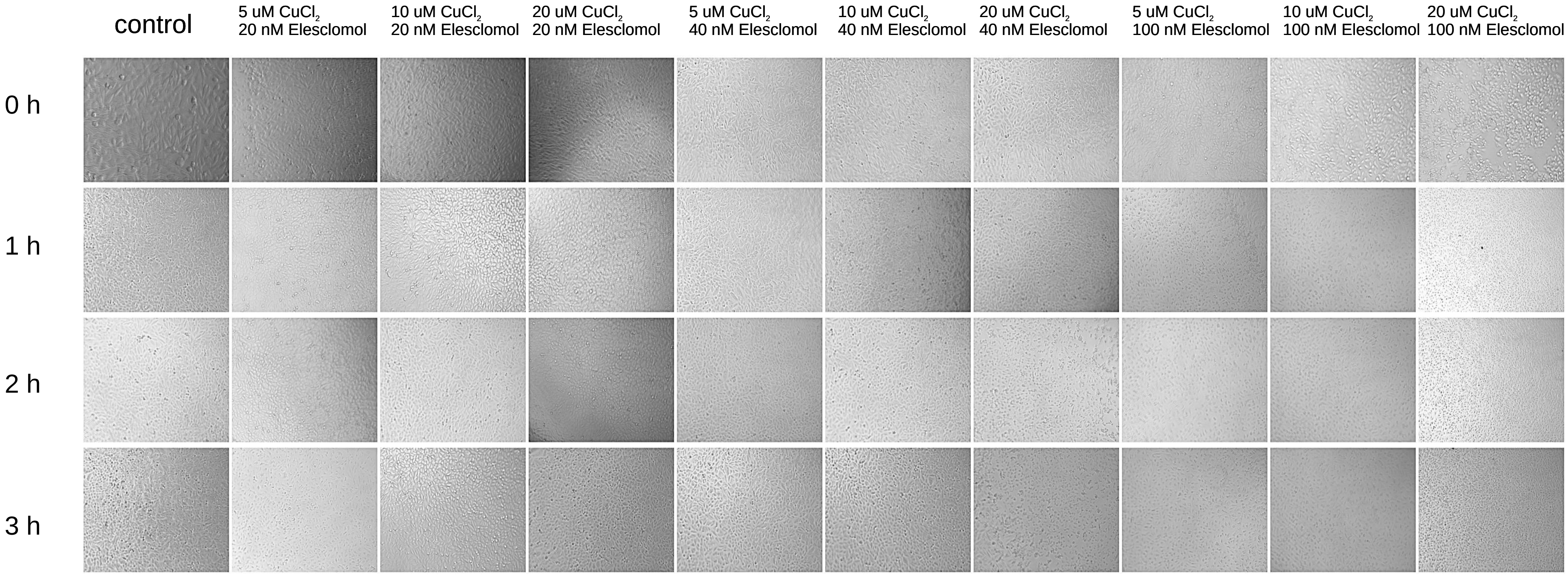

Figure 3.Status of LN229 at different drug concentrations and times.

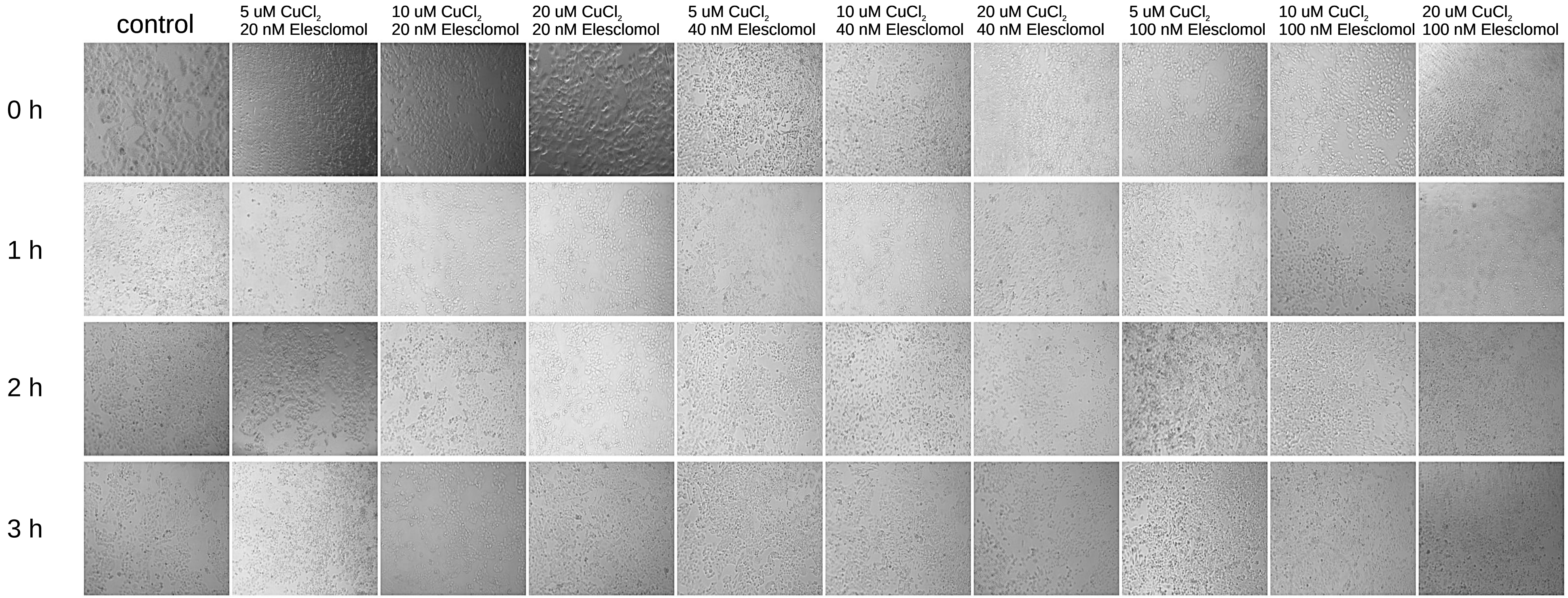

Figure 4.Status of U343 at different drug concentrations and times.
