# Supplementary material for: Cuproptosis-related lncRNAs and genes: Potential markers for glioblastoma prognosis and treatment
Source: PLoS One. 2025 Feb 6;20(2):e0315927. doi: 10.1371/journal.pone.0315927 (PMC11801720; doi:10.1371/journal.pone.0315927)

Supplementary Figure S3:
The session informationof R in this study.

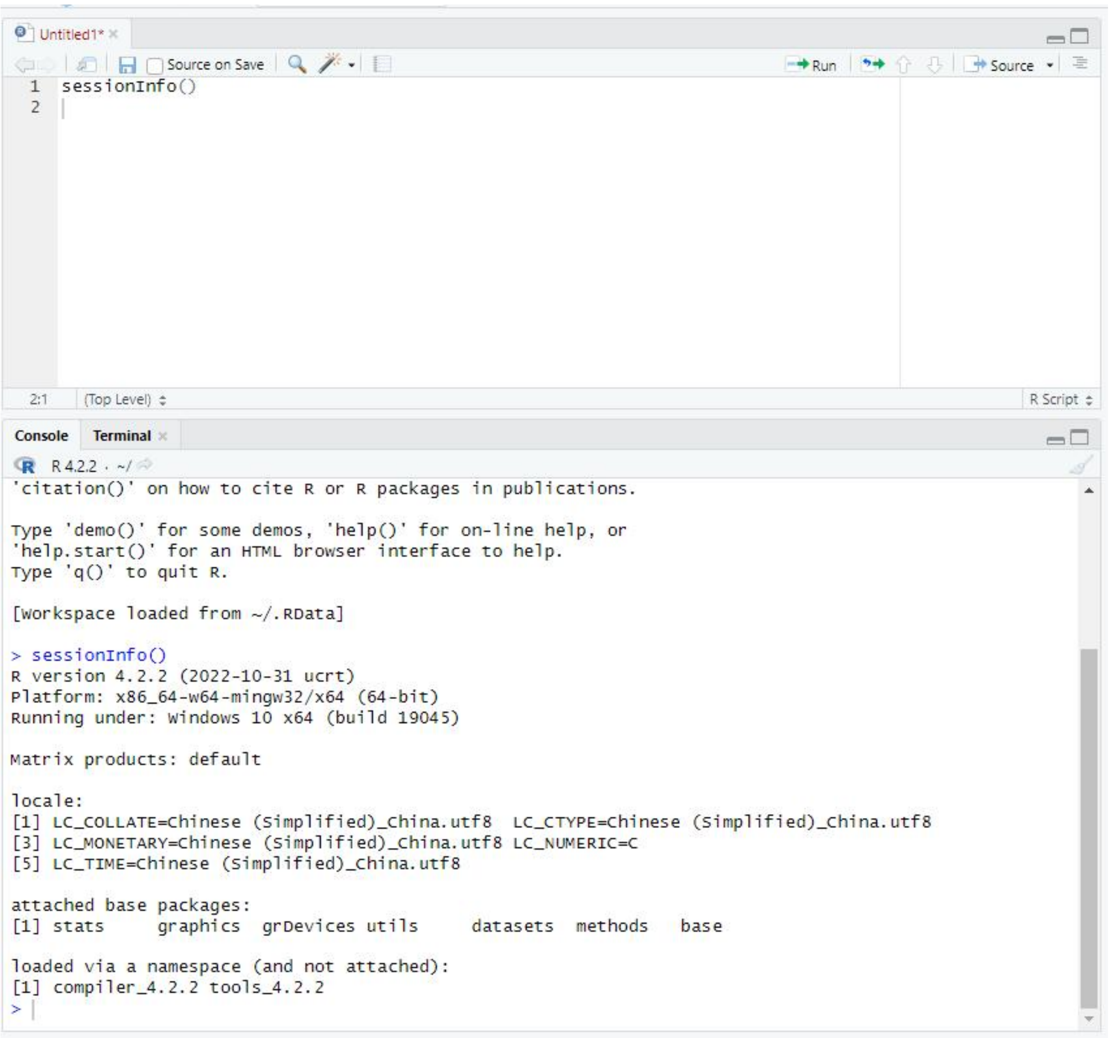

Supplement: S2 Fig — (PDF) [file pone.0315927.s002.pdf]
