# Supplementary material for: Cuproptosis-related lncRNAs and genes: Potential markers for glioblastoma prognosis and treatment
Source: PLoS One. 2025 Feb 6;20(2):e0315927. doi: 10.1371/journal.pone.0315927 (PMC11801720; doi:10.1371/journal.pone.0315927)
Supplement: S1 Table — (PDF) [file pone.0315927.s003.pdf]

Table 1 The clinical characteristics of patients in the TCGA database

Variable	
Gender	
Male/female/unknown	366/230/3
Age	
≤59/>59 /unknown	308/288/3
