# Supplementary material for: Cuproptosis-related lncRNAs and genes: Potential markers for glioblastoma prognosis and treatment
Source: PLoS One. 2025 Feb 6;20(2):e0315927. doi: 10.1371/journal.pone.0315927 (PMC11801720; doi:10.1371/journal.pone.0315927)
Supplement: S2 Table — (PDF) [file pone.0315927.s004.pdf]

Supplementary Table S1 | Sequences of primers used in this research

Targets	Forword5'-3'	Revers5'-3'
AC005229.4:lnc-EZH2-7	ACCACGGCTCACAACATAACA	TCCCAGAGCGGTACAGGAAT
AC091182.2:LINC01605	GGCGAGGACAAGAAGAGTTGA	CCCTTCGCTACCCACCAAAC
LIPT2	TGGCTCTCAACTGCTCTA	AGGTGGCATTACTTCTTCC
GLS	AGGGTCTGTTACCTAGCTTGG	ACGTTCGCAATCCTGTAGATTT
