# Supplementary material for: Cuproptosis-related lncRNAs and genes: Potential markers for glioblastoma prognosis and treatment
Source: PLoS One. 2025 Feb 6;20(2):e0315927. doi: 10.1371/journal.pone.0315927 (PMC11801720; doi:10.1371/journal.pone.0315927)
Supplement: S3 Table — (PDF) [file pone.0315927.s005.pdf]

Supplementary Table S2 | Multivariate Cox regression analysis

Id	coef
AC091182.2	0.382424336868827
AC005229.4	0.893936167468944
ZNF22-AS1	-0.658336798818582
