# Supplementary material for: Cuproptosis-related lncRNAs and genes: Potential markers for glioblastoma prognosis and treatment
Source: PLoS One. 2025 Feb 6;20(2):e0315927. doi: 10.1371/journal.pone.0315927 (PMC11801720; doi:10.1371/journal.pone.0315927)
Supplement: S1 Raw Images — (PDF) [file pone.0315927.s006.pdf]

S1_raw_images:

Western blotting of the cuproptosis cell model for three consecutive independent replicate experiments.

Cuproptosis-treated cells were collected and lysed on ice in RIPA buffer containing 1 mM protease inhibitor and phosphatase inhibitor for 40 min, followed by centrifugation at 12,000 rpm for 30 min at 4°C. The supernatants were collected and quantified by BCA protein assay (P0009, Beyotime, Shanghai, China). Protein (15 − 30 μg) was loaded onto SDS-PAGE gels and electrophoresed at 80 V for 15 min, followed by electrophoresis at 120 V for 40 min. The proteins were then transferred at 270 mA to a 0.22 μm PVDF membrane (Millipore, Tullagreen, Carrigtwohill, Ireland) for 72 min. The membranes were incubated with primary antibodies (FDX1, Abmart, T510671S, 1:1000; β-Tubulin, Abclonal, AC008, 1:1000) overnight in TBST containing 5% milk powder. Immunoblot imaging was performed using HRP-coupled secondary antibodies (HRP-labeled Goat Anti-Rabbit IgG (H+L), Beyotime, A0208, 1:1000) and ultra-high sensitivity ECL (BL520B, Biosharp, Anhui, China) with the MIni Chemi610 imager (SINSAGE, Beijing, China). The membranes were clipped to the appropriate target protein range prior to antibody incubation, and the strips were spliced back to the intact membrane state for exposure. Images of the pre-cut membranes and whole exposure membranes are shown in S1_raw_images.

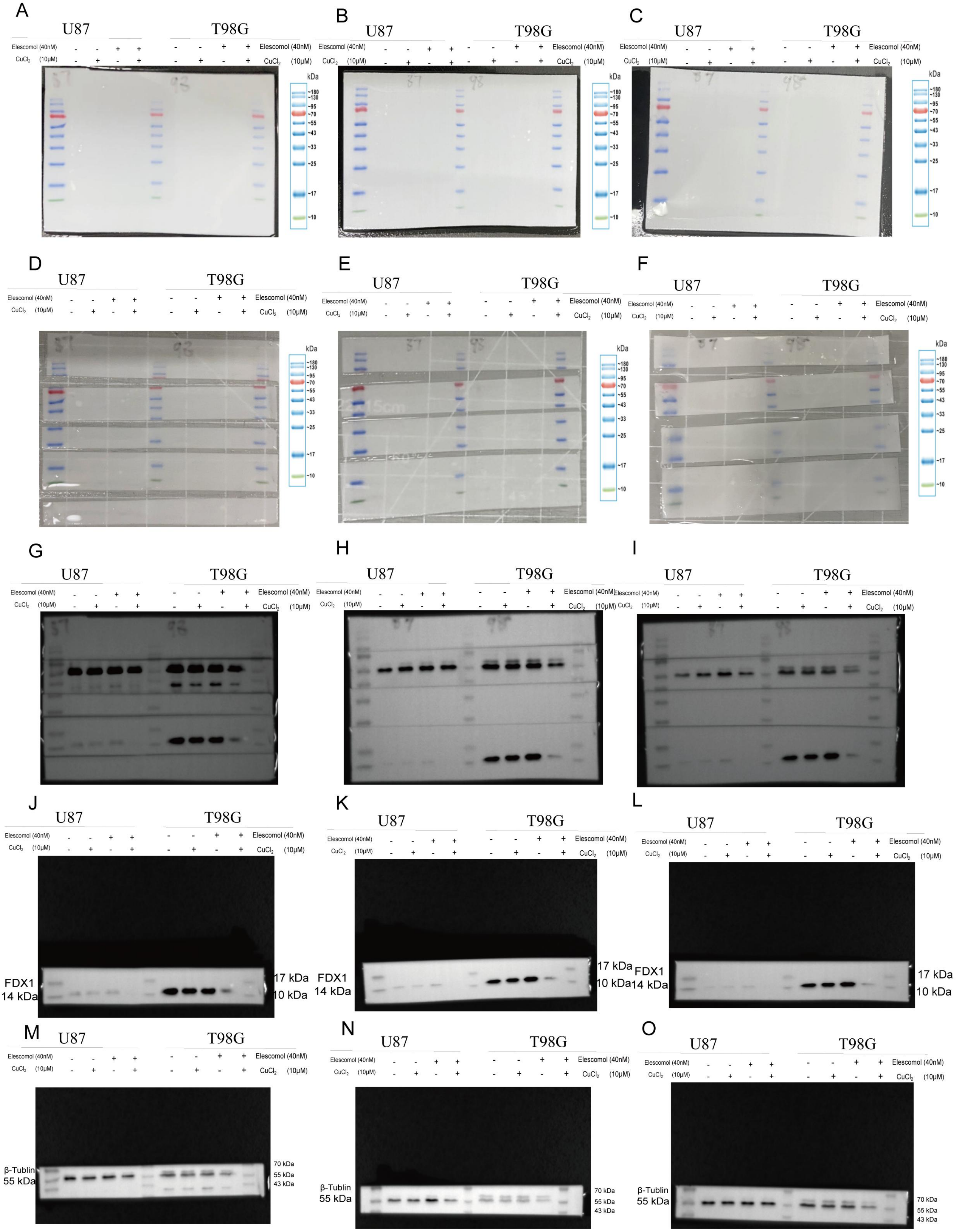

Figure1. Western blotting of the cuproptosis u87 (the left one) and T98G cell model (the right one) cell model)for three consecutive independent replicate experiments. (A-C) Images of membranes before clipping. (D-F) Images of clipped membranes. (G-I)The whole exposure film membrane image. (J-L) Single FDX1 image. (M-O) Single β-Tublin images.

The figure referenced in the manuscript is Fig 6A. The images within the manuscript are derived from Figure 1J, M.

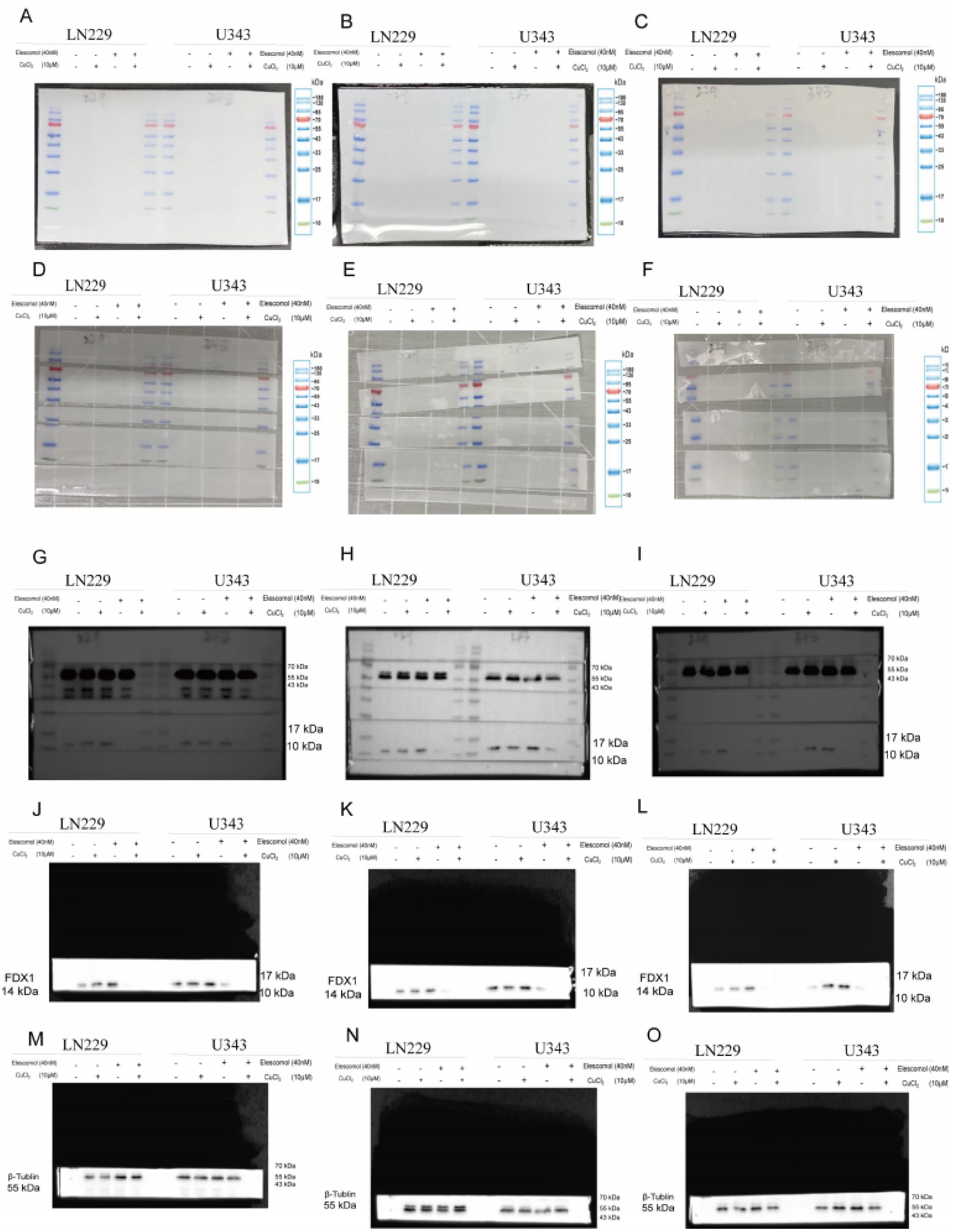

Figure2. Western blotting of the cuproptosis LN229 (the left one) and U343 cell model (the right one) cell model)for three consecutive independent replicate experiments.(A-C) Images of membranes before clipping. (D-F) Images of clipped membranes. (G-I)The whole exposure film membrane image. (J-L) Single FDX1 image. (M-O) Single β-Tublin images.

The figure referenced in the manuscript is Fig 6A. The images within the manuscript are derived from Figure 2G.

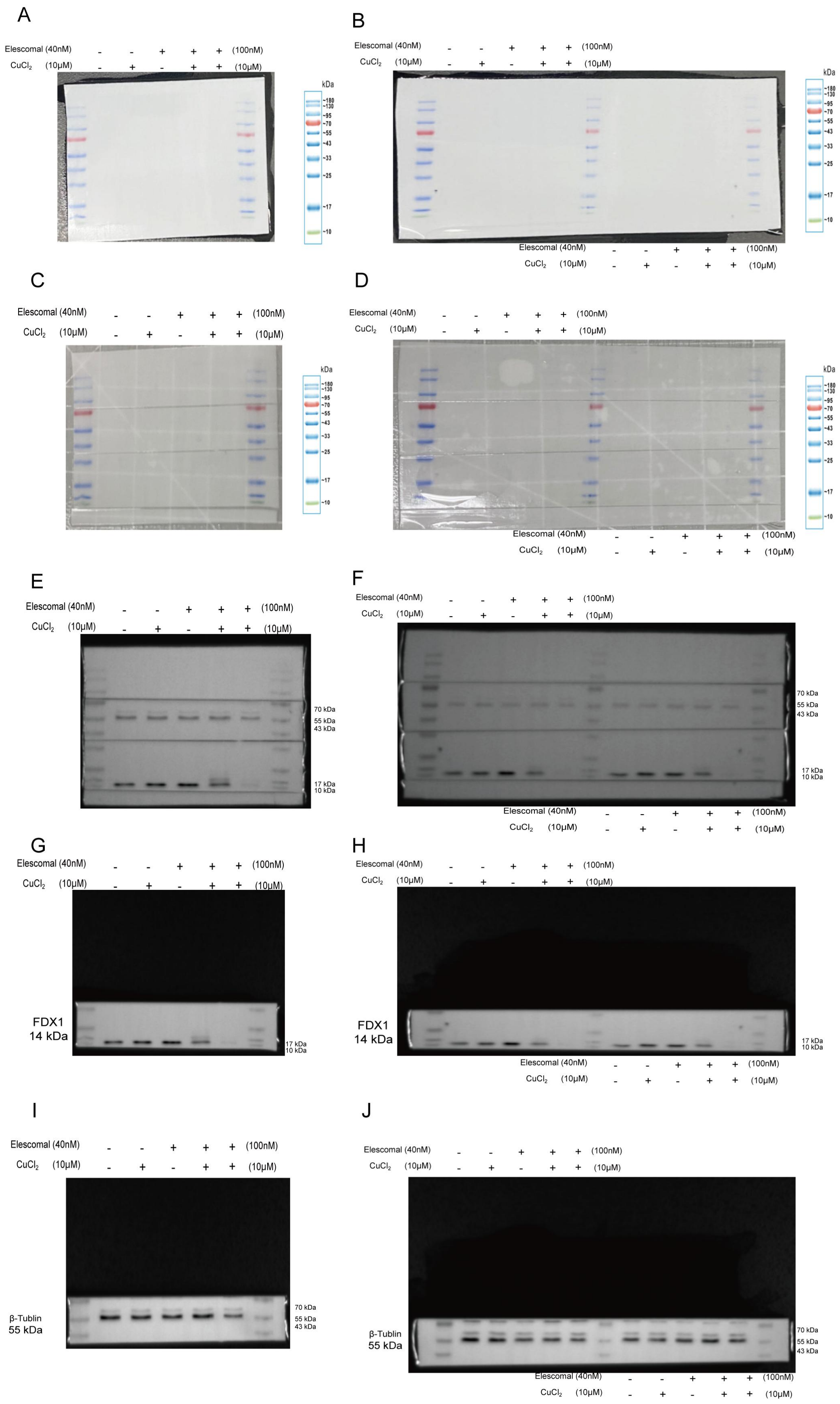

Figure3. Western blotting of the cuproptosis SVGP12 cell model for three consecutive independent replicate experiments. (A-B) Images of membranes before clipping. (C-D) Images of clipped membranes. (E-F) The whole exposure film membrane image. (G-H) Single FDX1 image. (I -J) Single β-Tublin images.

The figure referenced in the manuscript is Fig 7A. The images within the manuscript are derived from Figure 3G, I.
